# Temporary Setback or Lasting Challenge? Impact of Transient and Persistent Functional Disability on Wellbeing

**DOI:** 10.1093/geroni/igaf122.2172

**Published:** 2025-12-31

**Authors:** Thibault Kohler, Bram Vanhoutte

**Affiliations:** Universite Libre de Bruxelles, Brussels, Brussels Hoofdstedelijk Gewest, Belgium; Universite Libre de Bruxelles, Brussels, Brussels Hoofdstedelijk Gewest, Belgium

## Abstract

According to the hedonic treadmill theory, people’s wellbeing returns to a set-point after experiencing either positive or negative shocks. In the context of functional disability, studies differ on the degree of adaptation following this event. This study adds nuance to this debate by adopting a multidimensional approach to wellbeing in later life, differentiating between affective, cognitive, and eudemonic wellbeing, and examining how wellbeing trajectories are influenced by temporary functional disability, distinguishing between persistent and transient cases. We reordered longitudinal panel data from waves 4 to 8 of the Survey of Health, Ageing and Retirement in Europe (SHARE), from more than 3500 Europeans to align on the transition from no limitation to at least one limitation in ADL. We used linear spline growth models separately for each wellbeing measure (using Euro-D, Life Satisfaction and CASP) to examine non-linear trajectories. We observed a substantial decline in all three wellbeing measures at the onset of functional disability, with life satisfaction less affected (standardised mean differences = -0.11) than quality of life (-0.23) and depression (-0.27). On average, short-term disability led to a return to the initial level of wellbeing, while long-term disability led to a pronounced decline during the transition, with much less adaptation. Our findings highlight the significant impact that functional disability can have on wellbeing, revealing distinct patterns across various dimensions. Persistent disability often marks a crucial stage in the wellbeing of older people, whereas transient cases are characterised by a subsequent return to previous levels of wellbeing.

